# Intradermal Advanced Glycation End-products Relate to Reduced Sciatic Nerve Structural Integrity in Type 2 Diabetes

**DOI:** 10.1007/s00062-024-01493-1

**Published:** 2025-01-29

**Authors:** Christoph M. Mooshage, Dimitrios Tsilingiris, Lukas Schimpfle, Thomas Fleming, Stephan Herzig, Julia Szendroedi, Sabine Heiland, Martin Bendszus, Stefan Kopf, Felix Kurz, Johann Jende, Zoltan Kender

**Affiliations:** 1 Department of Neuroradiology, Heidelberg University Hospital, Heidelberg, Germany; 2 Department of Endocrinology, Diabetology, Metabolic Diseases and Clinical Chemistry (Internal Medicine 1), Heidelberg University Hospital, Im Neuenheimer Feld 410, 69120 Heidelberg, Germany; 3 Department of Neuroradiology, Division of Experimental Radiology, Heidelberg, Germany; 4 associated partner in the DZD, German Center for Diabetes Research, München-Neuherberg, Germany; 5 Helmholtz Diabetes Center, Helmholtz Center, Institute for Diabetes and Cancer (IDC), Munich, Neuherberg, Germany; 6 Joint Heidelberg-IDC Translational Diabetes Program, Innere Medicine 1, Heidelberg University Hospital, Heidelberg, Germany; 7 German Cancer Research Center, Heidelberg, Germany; 8 First Department of Internal Medicine, University Hospital of Alexandroupolis, Democritus University of Thrace, Alexandroupolis, Greece

**Keywords:** Advanced glycation end products, Skin autofluorescence, Diabetic neuropathy, Distal symmetric polyneuropathy, Magnetic resonance neurography, Diffusion tensor imaging

## Abstract

**Background:**

Cardiovascular risk management is beneficial, but stringent glycemic control does not prevent the progression of distal sensorimotor polyneuropathy (DSPN). Persistent hyperglycemia-induced alterations and cardiovascular factors may contribute to diabetes-associated nerve damage. This study aimed to evaluate the correlation between skin auto-fluorescence (sAF), an indicator of dermal advanced glycation end-product (AGE) accumulations, cardiovascular risk, and changes in peripheral nerve integrity.

**Methods:**

Sixty-two individuals with type 2 diabetes (T2D) (20 women and 42 men), including 29 diagnosed with DSPN (7 women and 22 men), and 10 healthy controls (HC) underwent diffusion tensor MR imaging of the sciatic nerve to assess fractional anisotropy (FA), an indicator of nerve structural integrity. sAF measurements were combined with clinical, serological, and electrophysiological evaluations. Arterial stiffness was assessed via pulse wave velocity (PWV).

**Results:**

sAF (HC 2.1 ± 0.25 AU, nDSPN 2.3 ± 0.47, DSPN 2.6 ± 0.43; *p* = 0.005) was higher in individuals with DSPN compared to HC (*p* = 0.010) and individuals without DSPN (*p* = 0.035). Within the group of T2D FA correlated negatively with sAF (r = −0.49, *p* < 0.001), PWV (r = −0.40, *p* = 0.009) and high-sensitivity troponin T (hsTNT), a marker of microvascular damage (r = −0.39, *p* < 0.001). In DSPN, sAF correlated positively with hsTNT (r = 0.58, *p* = 0.005) and with PWV (r = 0.52, *p* = 0.007), the sciatic nerve’s FA correlated negatively with PWV (r = −0.47, *p* = 0.010).

**Conclusions:**

This study is the first to show close correlations between reduced peripheral nerve integrity and both intradermal AGE deposition and arterial stiffness in individuals with T2D. These findings highlight a mechanistic link between glycation-related vascular injury and neuronal damage emphasizing the importance of cardiovascular risk management in preventing DSPN.

## Introduction

Despite extensive preclinical and clinical research on distal sensorimotor polyneuropathy (DSPN), the pathophysiology of one of the most common and most debilitating complications of diabetes mellitus remains incompletely understood [1]. As causal therapies for DSPN are still lacking, identification and risk stratification of DSPN are of major importance for individuals with diabetes. Hyperglycemia exerts detrimental effects on nerve function. While the control of cardiovascular risk factors is beneficial, tight glycemic control does not alleviate the progression of distal sensorimotor polyneuropathy (DSPN). Thus more sustained glycemia-induced modifications and cardiovascular factors might promote diabetes-related nerve damage. Advanced glycation end-products (AGEs) refer to a class of molecules formed by the non-enzymatic glycation of proteins, lipids and nucleic acids mainly build in hyperglycemic states but also driven by factors such as oxidative stress [2, 3]. Intracellular molecules modified by AGEs exhibit functional impairments and abnormal interactions with other proteins, while glycation of plasma proteins triggers overexpression and activation of surface receptors for AGEs (RAGE), thereby altering gene expression, and inducing proinflammatory responses. The intradermal deposition of AGEs has been established as a biomarker for cardiovascular complications that can be measured non-invasively via an AGE-reader that analyzes skin auto-fluorescence (sAF) by emitting [4, 5]. In diabetes mellitus, AGE accumulation in skin collagen correlates with both the duration and severity of hyperglycemia as well as with the presence of long-term complications [4].

Circulating AGEs are associated with diabetes-related microvascular complications such as DSPN it was shown that skin AGEs exhibit tighter correlations with diabetes related complications than circulating AGEs in individuals with type‑1 diabetes. Since blood and urine sampling do not necessarily reflect tissue AGE levels [6], the quantification of AGE accumulation in tissues provides a potential tool for assessing tissue damage and the risk of chronic complications. Meanwhile, it remains uncertain whether dermal AGE deposition is also associated with structural damage of distal and intraepidermal nerve fiber density or more proximally located nerve stems. [7–10]. However, multiple studies demonstrated associations of intradermal AGE deposition with clinical and electrophysiological parameters in individuals with T2D [7–9, 11] and studies applying MRN demonstrated proximal predominance of peripheral nerve damage [12]. Aside from that, cardiovascular risk factors are known to be importance in the development of DSPN [13]. Pulse wave velocity (PWV) represents a broadly available marker for aortic stiffness and correlates with macrovascular dysfunction in individuals with type 2 diabetes [14, 15], yet, it remains to be investigated whether both factors are associated with the peripheral nerves’ structural integrity in individuals with type 2 diabetes.

Novel imaging techniques such as diffusion tensor imaging (DTI) allow for an accurate analysis of peripheral nerves’ structural integrity in individuals with diabetes [16, 17]. Studies of DTI MRN, and specifically on the nerves’ fractional anisotropy (FA) as a marker of the nerve’s structural integrity, found that levels of high-sensitivity troponin T (hsTNT), a marker of microvascular complications in T2D, reflect the degree of structural nerve damage in individuals with T2D [14, 15, 18].

Since up to date data on the associations of structural changes of the sciatic nerve with dermal AGE deposition in individuals with T2D are lacking, this study aimed to investigate whether dermal AGE accumulation, driven by prolonged hyperglycemia and cardiovascular risk, is associated with changes in nerve structural integrity. Relationships were assessed between peripheral nerve structure, measured by diffusion tensor imaging (DTI) magnetic resonance neurography, intradermal AGE deposition (via skin autofluorescence), and vascular function indicators like arterial stiffness and hsTNT.

## Materials and Methods

### Study Design

The local ethics committee of Heidelberg university hospital approved this prospective study (HEIST-DiC, clinicaltrials.gov identifier NCT03022721, local ethics number S‑383/2016) and all participants gave written informed consent. The Department of Endocrinology, Diabetology, Metabolic Diseases and Clinical Chemistry (Internal Medicine 1) of the Heidelberg University Hospital conducted the screening, recruitment, and the instrument-based and clinical examinations of all participants between January 2016 and November 2019. Exclusion criteria were defined in analogy to previously published MRN studies involving individuals with T2D [19]. Intradermal accumulation of AGEs was measured non-invasively on a 4 cm^2^ area of the volar forearm via an AGE Reader (DiagnOptics BV, Netherlands) which emits ultraviolet light to determine the sAF, which is indicative of intradermal AGE deposition. sAF was then calculated by dividing the average emitted light intensity by the average extinction light intensity multiplied by 100. Results were expressed in arbitrary units (AU) [20, 21]. To assure a precise measurement of sAF, individuals with tattoos, suffering from dermatologic diseases or who were exposed to skin care creams or other substances with fluorescent properties were excluded from this study. To minimize potential confounding factors of structural nerve integrity groups were matched for age, sex, body mass index (BMI) and estimated glomerular filtration rate (eGFR).

A post hoc power analysis, based on a previously published study investigating associations of DTI-MRN parameters with clinical and serological variables [18] yielding a power of 0.79, was performed to justify the cohort size of this study. Subsequently, for an α level of 0.05, a power of 0.8 and an effect size of 0.4, the latter as suggested by Cohen to identify large effect sizes [22], a total sample size of 66 participants was required to conduct one-way ANOVA to compare three groups.

A standard serological testing and an additional hsTNT assay were performed in fasting state. To calculate the eGFR Cystatin C values were analyzed in each patient.

Neurological assessment included testing of Neuropathy Symptom Score (NSS) and Neuropathy Disability Score (NDS) as well as nerve conduction studies on the patient’s right including the measurement of nerve conduction velocities (NCVs) of tibial, peroneal, and sural nerves, sensory nerve action potentials (SNAPs) of the sural nerve and compound muscle action potentials (CMAPs) of the tibial and peroneal nerve. DSPN was diagnosed upon existence if at least one of the following three criteria was met: *(1)* a score of ≥ 6 in NDS, *(2)* a score of 3–5 in NDS *and* a score of 5–6 in NSS [23] and/or *(3)* abnormal results of nerve conduction parameters in one nerve and signs or symptoms of DSPN [24].

Furthermore, intradermal AGE levels were measured via sAF at the volar surface of the forearm using an AGE Reader (SU, DiagnOptics BV, Netherlands), as described in detail previously [20]. As several exogenous factors may influence the measurement of sAF, most prominently smoking but also food, individuals underwent all examinations in a fasting state [7]. The pulse wave velocity (PWV), a measure of arterial stiffness, was determined through non-invasive blood pressure measurements of the arms and ankles (ABI System 1000; Boso d.o.o.) [14, 15, 20]. Immediately thereafter individuals were transferred to the department of Neuroradiology to undergo MRN, as outlined below.

### MRI Imaging Protocol and Data Analysis

#### Magnetic Resonance Neurography Imaging Protocol

High-resolution MRN of the right leg was performed in a 3.0 T MR-scanner (Magnetom TIM-TRIO, Siemens, Erlangen, Germany) using a 15-channel transmit-receive extremity coil. The sequence protocol consisted of axial high resolution T2-weighted (T2w) turbo spin echo 2D sequence with spectral fat saturation of the right mid-thigh (A) and an DTI with an axial fat-suppressed, diffusion-weighted two-dimensional echo-planar sequence with the following parameters:

- Repetition time (TR) 5970 ms, echo time (TE) 55 ms, field of view (FOV) 160 × 160 mm2, matrix size 512 × 512, slice thickness 4 mm, interslice gap = 0.35 mm, voxel size 0.3 × 0.3 × 4.0 mm3, 3 averages, 24 images.
- TR = 5100 ms; TE = 92.8 ms; b = 0 and 1000 s/mm2; directions = 20; FOV = 160 × 160 mm2; matrix size = 128 × 128; slice thickness = 4 mm; voxel size = 1.3 × 1.3 × 4 mm3; no interslice gap, 3 averages, 24 slices, 1512 images.

### Image Post-processing

After pseudonymization of the images, post-processing was conducted by two trained neuroradiologists with 3 and 7 years of experience in MRN imaging. Nordic BRAINEX (NordicNeuroLab AS, 2019), a software designed for automated calculation and reconstruction of fiber tracts in diffusion-weighted imaging, was then used for the analysis of the DTI sequence to determine the sciatic nerve’s fractional anisotropy (FA), as performed and explicitly described before [20, 25]. The axial T2w sequence (A) was used for automatic co-registration with the DTI sequence (B). After manual delineation of the anatomic region of the sciatic nerve, the latter was automatically tracked. We chose a value of > 0.1 for the nerve’s FA, a maximum tract turning angle of 41.4 degrees, a minimum fiber length of 20 mm and one seed per voxel as cut-off values for automated fiber tracking, as performed before [20].

**Fig. 1:**
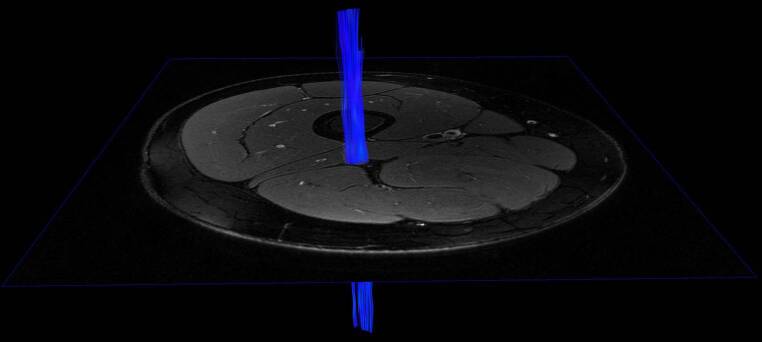
Three-dimensional reconstruction of nerve fiber tracking using diffusion tensor imaging of the right sciatic nerve in an individual with type 3 diabetes without diabetic neuropathy

Nordic BRAINEX then automatically determined the sciatic nerve’s average FA, which is a dimensionless parameter with values ranging between 0 and 1. The FA is known to be a measure of the structural integrity of peripheral nerves [20] being correlated with electrophysiological markers of axonal and myelin sheath integrity [18, 20]. Fig. 1 displays a reconstructed, 3‑dimensional fiber track of the right sciatic nerve in an individual with type 2 diabetes without DSPN.

### Statistical Analysis

GraphPad Prism 9 (GraphPad Software, La Jolla, CA, USA) and SPSS 28 (IBM, Armonk, NY, USA) were used for all statistical analyses. Differences between categorial variables were tested applying the Chi-Square test. of Subject to the pattern of data distribution T‑tests or Mann-Whitney-tests were used to compare two groups and one-way ANOVAs with post-hoc Tukey tests or the Kruskal-Wallis test with post-hoc Dunn tests were applied for comparisons of more than two groups. Likewise, Pearson or Spearman correlation coefficients were applied for correlation analyses. If multiple significant correlations were found for one parameter, we performed partial correlation analyses to preclude confounding. Additionally, we performed receiver operating characteristic (ROC) curve analysis to analyze the diagnostic value of sAF, FA and both taken together regarding DSPN. All results are presented as mean values ± standard deviation.

## Results

### Group Comparisons of All Compiled Data

10 healthy controls (HC) and 62 individuals with T2D (20 women, 42 men) took part in this study. 29 individuals with T2D were diagnosed with DSPN (7 women, 22 men) while 33 individuals (13 women, 20 men) did not show signs and symptoms of DSPN (nDSPN). No differences for age, sex distribution, BMI, eGFR were found between the three groups. Furthermore, there was no difference in duration of T2D between individuals with and without DSPN. A detailed summary of all group comparisons, including electrophysiological, clinical and serological parameters, can be found in Table 1.

**Table 1:** Group comparisons of demographic, serologic, electrophysiological and diffusion tensor imaging parameters of the sciatic nerve of all study participants

	HC	nDSPN	DSPN	*p*-value	HC vs. nDSPN	HC vs. DSPN	nDSPN vs. DSPN
FA	0.467 ± 0.056	0.446 ± 0.064	0.407 ± 0.048	0.005^A^	0.582	0.015	0.022
Age (years)	61.3 ± 6.1	62.0 ± 9.5	66.3 ± 7.2	0.077^A^	0.972	0.223	0.099
Gender (f/m)	5/5	13/20	7/22	0.249^X^	n. a.	n. a.	n. a.
BMI (kg/m^2^)	27.8 ± 3.5	29.2 ± 4.3	29.9 ± 4.4	0.423^A^	0.650	0.394	0.804
Diabetes duration (years)	n. a.	8.6 ± 6.2	12.5 ± 8.8	n. a.	n. a.	n. a.	0.051^T^
NDS	0.5 ± 1.3	1.4 ± 1. 3	6.1 ± 1.9	<0.001^K^	0.628	<0.001	<0.001
NSS	0.0 ± 0.0	2.6 ± 3.1	5.6 ± 2.7	<0.001^K^	0.111	<0.001	0.001
Sural nerve NCV (m/s)	44.8 ± 4.3	47.9 ± 6.4	42.7 ± 5.6	0.051^A^	0.373	0.720	0.050
Sural nerve SNAP (µV)	8.0 ± 6.5	6.4 ± 3.4	5.1 ± 4.1	0.130^K^	>0.999	0.322	0.208
Peroneal nerve NCV (m/s)	43.1 ± 3.2	42.2 ± 5.3	38.6 ± 5.8	0.016^A^	0.892	0.073	0.029
Peroneal nerve CMAP (mV)	7.5 ± 2.9	6.5 ± 3.7	4.2 ± 2.8	0.010^K^	0.611	0.018	0.087
Peroneal nerve DML (ms)	4.1 ± 0.5	5.2 ± 2.6	6.2 ± 3.6	0.182^K^	>0.999	0.286	0.565
Tibial nerve NCV (m/s)	44.6 ± 6.5	42.6 ± 4.9	38.4 ± 4.6	0.001^A^	0.554	0.006	0.006
Tibial nerve CMAP (mV)	11.6 ± 7.6	14.0 ± 5.7	8.1 ± 6.1	0.002^A^	0.551	0.310	0.001
Tibial nerve DML (ms)	5.5 ± 3.6	5.1 ± 3.2	6.6 ± 4.7	0.118^K^	0.720	>0.999	0.134
Glucose (mg/dl)	93.5 ± 7.1	152.3 ± 42.5	138.3 ± 29.4	<0.001^K^	<0.001	<0.001	0.979
HbA1c (mmol/mol)	33.1 ± 5.6	55.1 ± 14.4	52.9 ± 11.6	<0.001^K^	<0.001	<0.001	>0.999
GFR (ml/min)	93.8 ± 18.9	86.1 ± 16.6	84.0 ± 14.0	0.226^K^	0.513	0.255	>0.999
sAF (AU)	2.1 ± 0.3	2.3 ± 0.5	2.6 ± 0.4	0.005^A^	0.417	0.010	0.035
PWV (ms)	12.6 ± 1.4	13.3 ± 1.9	13.6 ± 1.7	0.284^A^	0.535	0.535	0.320
hsTNT (pg/mL)	6 ± 1.7	8.5 ± 4.7	11.7 ± 5.7	0.003^K^	0.269	0.004	0.068

All values are displayed as mean ± standard deviation

*f* female; *m* male; *HC* healthy controls; *DSPN* distal sensorimotor polyneuropathy; *FA* fractional anisotropy; *BMI* Body-mass index; *n.* *a.* not applicable; *NDS* Neuropathy disability score; *NSS* Neuropathy severity scale; *NCV* nerve conduction velocity; *CMAP* compound motor action potential; *SNAP* sensory nerve action potential; *GFR* glomerular filtration rate; *sAF* skin auto-fluorescence; *AU* arbitrary units

^M^*p*-value obtained from Mann-Whitney U test

^T^*p*-value obtained from T‑Test

^A^*p*-values obtained from ANOVA with post-hoc Tukey test for multiple comparison

^K^*p*-value obtained from Kruskal-Wallis test with post-hoc Dunn test for multiple comparison

^X^*p*-value obtained from Chi-Square test

sAF (HC 2.1 ± 0.25 AU, nDSPN 2.3 ± 0.47, DSPN 2.6 ± 0.43; *p* = 0.005) was 23.8% higher in individuals with DSPN compared to HC (*p* = 0.010) and 9.5% higher in individuals without DSPN (*p* = 0.035). hsTNT (HC 6.0 ± 1.7 pg/ml, nDSPN 8.5 ± 4.7 pg/ml, DSPN 11.7 ± 5.7 pg/ml; *p* = 0.003) was higher in individuals with DSPN compared to HC (*p* = 0.004). No differences could be found for PWV between the groups.

FA (HC 0.47 ± 0.06, nDSPN 0.45 ± 0.06, DSPN 0.41 ± 0.05; *p* = 0.005) was lower in DSPN individuals compared to HC (*p* = 0.015) and individuals without DSPN (*p* = 0.022). NDS (HC 0.5 ± 1.3, nDSPN 1.4 ± 1. 3, DSPN 6.1 ± 1.9; *p* < 0.001) and NSS (HC 0.0 ± 0.0, nDSPN 2.6 ± 3.1, DSPN 5.6 ± 2.7; *p* < 0.001) were higher in individuals with DSPN compared to HC (*p* < 0.001;) and individuals without DSPN (*p* < 0.001 and *p* = 0.001, respectively).

### Correlation Analyses of the Sciatic Nerve’s FA

In HC, FA correlated positively with sural SNAP (r = 0.75, *p* = 0.025) and peroneal (r = 0.84, *p* = 0.005) and tibial NCV (r = 0.68, *p* = 0.045).

**Fig. 2:**
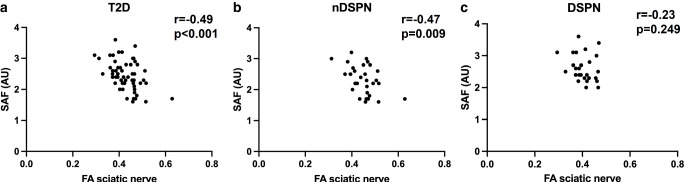
Correlations of the sciatic nerve’s fractional anisotropy (FA) with skin auto-fluorescence (sAF) in arbitrary units (AU) in individuals with diabetes type 2 (T2D) with (DSPN) and without distal sensorimotor diabetic polyneuropathy (nDSPN). **a** Correlation of the sciatic nerve’s FA with sAF in individuals with T2D (r = −0.49; *p* < 0.001) and (**b**) in individuals without DSPN (r = −0.47; *p* = 0.009). **c** No such correlation was found in T2D individuals with DSPN (r = −0.23; *p* = 0.249)

In correlation analyses over all T2D FA correlated positively with GFR (r = 0.39, *p* = 0.033) and negatively with sAF (r = −0.49, *p* < 0.001; Fig. 2a), PWV (r = −0.40, *p* = 0.009) and hsTNT (r = −0.39, *p* < 0.001). Partial correlation analysis of the sciatic nerve’s FA controlled for eGFR and age confirmed a negative correlation with sAF (r = −0.30, *p* = 0.039). Partial correlation analyses controlled for eGFR yielded negative correlations of the sciatic nerve’s FA with NDS (r = −0.52; *p* < 0.001), peroneal (r = −0.36, *p* = 0.013) and tibial DML (r = −0.35, *p* = 0.017), PWV (r = −0.32, *p* = 0.028) and hsTNT (r = −0.41, *p* = 0.004). Further partial correlation analyses controlled for eGFR confirmed positive correlations with peroneal NCV (r = 0.55, *p* < 0.001) and CMAP (r = 0.57, *p* < 0.001), tibial NCV (r = 0.62, *p* < 0.001) and CMAP (r = 0.44, *p* = 0.002).

In individuals without DSPN, we found a positive correlation with eGFR (r = 0.39, *p* = 0.043) and negative correlations with sAF (r = −0.47, *p* = 0.009; Fig. 2b) and hsTNT (r = −0.54, *p* = 0.003). Partial controlled correlation analysis of the sciatic nerve’s FA with sAF controlled for GFR and age resulted in a stable trend of a negative correlation (r = −0.36, *p* = 0.075).

In individuals with DSPN we found a negative correlation of the sciatic nerve’s FA with PWV (r = −0.47, *p* = 0.011). No correlation of the sciatic nerve’s FA with sAF was found for individuals with DSPN (r = −0.23, *p* = 0.249; Fig. 2c).

A detailed summary of the correlation analysis of the sciatic nerve’s FA with a compiled data, including nerve conduction studies and NSS and NDS, can be found in Table 2**.**

**Table 2:** Correlations of the sciatic nerve’s fractional Anisotropy of all study participants with demographic, instrumental-based, clinical and serological parameters

	HC		T2D		nDSPN		DSPN	
	r	*p*	r	*p*	r	*p*	r	*p*
Age (years)	0.1	0.776^P^	−0.21	0.103^P^	−0.19	0.292^P^	−0.03	0.865^P^
BMI (kg/m^2^)	0.29	0.409^P^	−0.30	0.611^P^	0.01	0.974^P^	−0.12	0.552^P^
Diabetes duration (years)	n. a.	n. a.	0.21	0.863^P^	0.07	0.682^P^	0.17	0.403^P^
NDS	0.03	0.956^S^	−0.46	<0.001^S^	−0.32	0.068^P^	−0.18	0.363^P^
NSS	n. a.	n. a.	−0.15	0.249^S^	−0.14	0.452^S^	0.18	0.343^P^
Sural nerve NCV (ms)	0.54	0.129^P^	0.09	0.598^S^	0.17	0.394^P^	−0.34	0.301^P^
Sural SNAP	0.75	0.025^S^	0.27	0.073^S^	0.25	0.195^P^	−0.11	0.671^S^
Peroneal nerve NCV	0.84	0.005^P^	−0.24	<0.001^P^	0.41	0.024^P^	0.42	0.027^P^
Peroneal nerve CMAP	0.13	0.763^P^	0.59	<0.001^S^	0.58	<0.001^S^	0.55	0.002^S^
Peroneal nerve DML	0.15	0.732^P^	−0.37	0.004^S^	−0.47	0.008^S^	−0.32	0.098^S^
Tibial nerve NCV	0.68	0.045^P^	−0.23	<0.001^P^	0.44	0.014^P^	0.53	0.004^P^
Tibial nerve CMAP	0.12	0.764^P^	0.45	<0.001^S^	0.19	0.318^P^	0.50	0.007^P^
Tibial nerve DML	0.22	0.561^P^	−0.32	0.013^S^	−0.21	0.253^S^	−0.34	0.075^S^
Glucose (mg/dl)	−0.26	0.463^P^	0.01	0.916^S^	−0.02	0.917^P^	−0.18	0.339^S^
HbA1c (mmol/mol)	−0.05	0.884^P^	−0.09	0.499^S^	−0.12	0.502^S^	−0.01	0.955^S^
GFR (ml/min)	0.67	0.076^S^	0.39	0.033^P^	0.39	0.043^P^	0.11	0.614^P^
sAF (AU)	−0.36	0.347^P^	−0.49	<0.001^P^	−0.47	0.009^P^	−0.23	0.249^P^
PWV (m/s)	0.33	0.391^P^	−0.40	0.009^S^	−0.22	0.240^S^	−0.47	0.011^P^
hsTNT	0.17	0.688^P^	−0.39	<0.001^S^	−0.54	0.003^S^	−0.40	0.062^S^

*HC* healthy controls; *DSPN* distal sensorimotor polyneuropathy; *BMI* Body-mass index; *n.* *a.* not applicable; *NDS* Neuropathy disability score; *NSS* Neuropathy severity scale; *NCV* nerve conduction velocity; *CMAP* compound motor action potential; *SNAP* sensory nerve action potential; *GFR* glomerular filtration rate; *sAF* skin auto-fluorescence; *AU* arbitrary units

^P^*p*-value obtained from Pearson correlation analysis

^S^*p*-value obtained from Spearman correlation analysis

### Correlation Analysis of SAF

In individuals with T2D, sAF correlated positively with age (r = 0.49, *p* < 0.001), NDS (r = 0.28, *p* = 0.038), peroneal CMAP (r = −0.37, *p* = 0.007), PWV (r = 0.42 = 0.002) and hsTNT (r = 0.39, *p* = 0.005) while a negative correlation with tibial NCV (r = −0.31, *p* = 0.022) was found. Of these, correlations with peroneal CMAP (r = −0.35, *p* = 0.011) and PWV (r = 0.36, *p* = 0.008) remained significant in partial correlation analysis controlled for age.

In individuals without DSPN, sAF correlated positively with age (r = 0.50, *p* = 0.005) and negatively with sural SNAP (r = −0.42, *p* = 0.035) and peroneal CMAP (r = −0.40, *p* = 0.031), which both retained a trend of correlation in partial correlation analysis controlled for age, yet without reaching statistical significance (sural SNAP: r = −0.34, *p* = 0.095; peroneal CMAP: r = 0.35, *p* = 0.067).

In individuals with DSPN, sAF correlated positively with hsTNT (r = 0.58, *p* = 0.005) and with PWV (r = 0.52, *p* = 0.007), which both remained significant in a partial correlation analysis controlled for the sciatic nerve’s FA (hsTNT: r = 0.51, *p* = 0.017; PWV: r = 0.43, *p* = 0.034).

### ROC Analysis

**Fig. 3:**
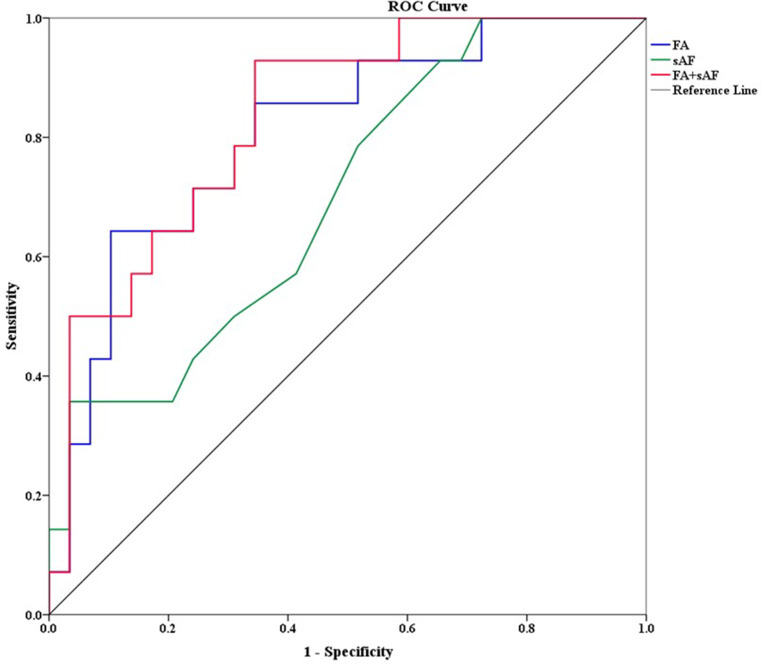
Receiver operating characteristic (ROC) curve for skin auto-fluorescence (sAF; green line), fractional anisotropy (FA; blue curve) and both parameters taken together (red curve). The area under the curve (AUC) was 69.7% for sAF, 80.8% for FA alone and 83.3% for both parameters taken together

To investigate the diagnostic validity of sAF and FA as a marker for DSPN, we performed ROC analysis for sAF and FA separately and taken together against the criteria to diagnose DSPN as issued above: For sAF alone, the area under the curve (AUC) was 69.7% (95% c.i. 53.4–86.0%), for FA alone 80.8% (95% c.i. 67.0–94.6%). A FA value <0.39 showed a 64.3% sensitivity and a 89.7% specificity (Youden Index 0.54) while a FA value <0.43 had a sensitivity and specificity of 85.7 and 65.5% respectively (Youden Index 0.50) for the presence of DSPN. sAF values <2.0 AU essentially excluded the presence of DSPN (sensitivity 100%, specificity 37.6%, negative predictive value 100%), while those >3.0 were highly suggestive of DSPN (sensitivity 35.7%, specificity 96.6%, positive predictive value 85.0%). The combination of both parameters taken together showed slightly better characteristics than FA alone, with a ROC-AUC of 83.3% (95% c.i. 70.9–95.6%) (Fig. 3).

## Discussion

This study combines in vivo DTI of the sciatic nerve to assess the structural integrity of proximal peripheral nerves, as measured by the fractional anisotropy, in conjunction with skin autofluorescence as a surrogate for dermal AGE deposition and serological and electrophysiologic measures to investigate the influence of glycation-related vascular injury on nerve damage in T2D. The main findings were that (I) in individuals with T2D and DSPN the sciatic nerve’s FA was lower while sAF was higher compared to HC and individuals without DSPN, respectively. (II) Over all individuals suffering from T2D the sciatic nerve’s FA correlated negatively with sAF, PWV, and hsTNT independently of confounding factors age and GFR. (III) In individuals with DSPN, sAF correlated positively with hsTNT and PWV while the sciatic nerve’s FA correlated negatively with PWV.

Previous studies found correlations of sAF with electrophysiological parameters and clinical scores of diabetic neuropathy and could further demonstrate that sAF is an independent predictor of DSPN in T2D [21, 26]. In T1D, it could be shown that several pathways of AGE formation are involved in the development of DSPN [27]. However, several studies demonstrated that glucose control is beneficial in individuals with T1D, yet, has no substantial effect on the prevention and progression of DSPN in individuals with T2D [28, 29]. These results indicate that a combination of hyperglycemia and hyperinsulinemia is of pathophysiological relevance in T2D. Furthermore, the results suggest that a persisting glucose-induced modification of proteins causes structural damage and dysfunction of peripheral nerves in individuals with T2D, arising the question to what extent AGE deposition relates to structural nerve damage of peripheral nerves in vivo. This study demonstrates associations between intradermal AGE deposition and the structural integrity of proximal peripheral nerves in individuals with T2D in vivo, suggesting that AGE accumulation may contribute to peripheral nerve damage. However, since ethical considerations did not allow to obtain specimens of peripheral nerves our results do not allow to outline a causal relationship between skin AGEs and peripheral nerve damage. Nevertheless, it has previously been suggested that an accumulation of intradermal AGEs may precede the progression of DSPN [30]. This hypothesis is supported by longitudinal studies that found sAF to be an independent predictor for the development of DSPN in individuals with T2D [26]. Further, studies on experimental diabetic neuropathy have shown that the interaction of AGEs with the receptor for AGEs may play a major role in the pathogenesis of DSPN [31, 32] suggesting that the formation of AGEs is one of the main drivers of diabetes-related complications in individuals with T2D. [7]. Nonetheless, elevated level of skin AGEs is associated with the severity of diabetes complications in general and may therefore serve as an indicator of hyperglycemic stress which is associated with peripheral nerve damage [2].

Since intradermal AGE deposition is also associated with the risk of developing cardiovascular complications, as represented by its correlation with the Framingham risk score [33, 34], and optimizing cardiovascular risk factors was shown to reduce the risk of DSPN [13], it is of particular interest to investigate the association micro- and macrovascular parameters such as hsTNT and PWV with intradermal AGE deposition and the structural integrity of peripheral nerves. The finding that over all T2D individuals sAF was correlated with PWV, independently of confounding factors, and that the sciatic nerve’s FA correlated negatively with sAF, PWV and hsTNT, may imply that an increased AGE deposition in the skin is associated with diabetic angiopathy and thereby impacting the peripheral nerves’ microcirculation and subsequently lead to nerve damage [2, 18, 26, 35]. Interestingly, the negative correlation between FA and PWV in individuals with DSPN was even stronger as compared to the correlation found over all T2D individuals while no such correlation was found in individuals without DSPN: Taken together with the findings that hsTNT and sAF were higher in DSPN individuals compared to HC and DSPN, which was confirmed by previous studies [18, 30], it may be hypothesized that measures of sAF, PWV and hsTNT, which are known markers of vascular dysfunction in T2D [20, 35], aggravate or accumulate during the disease process and thereby contribute to the decrease of the nerve’s structural integrity and concomitant nerve damage [18, 20, 35]. This assumption is supported by the fact that sAF was correlated with hsTNT and PWV in individuals with DSPN [36]. While these results underline the importance of modifying cardiovascular risk factors that can be adjusted to prevent the onset and progression of DSPN in individuals with T2D, managing these risk factors remains insufficient [37].

In contrast to the correlation between the sciatic nerve’s FA and individuals’ sAF values we could not find any association of FA with values of HbA1c. This finding may be explained by the fact that HbA1c values are subject to fluctuations while sAF embodies a parameter of continuous and cumulative AGE deposition in hyperglycemic states and that the impact of HbA1c on the nerve’s microvasculature and thereby to nerve damage differs between individuals with T2D with and without DSPN [38].

Apart from the strong discriminative power of FA for DSPN, ROC analysis also yielded sAF cut-off values with excellent negative and positive predictive values for the presence of DSPN (3.0 and 2.0, respectively). Combining sAF with FA only slightly improved the diagnostic features of FA alone with respect to presence of DSPN.

Preclinical studies have identified numerous potential mechanisms and effective treatments for DSPN. However, most trials on substances proposed to reduce AGE accumulation in DSPN outcomes employed aldose reductase (AR) inhibitors or B vitamins, with varying results [39]. Epalrestat, an AR inhibitor showed efficacy in preserving nerve function and alleviating symptoms in an open-label trial, while a vitamin b1 derivate benfotiamine exhibited symptom reduction in a six-week randomized controlled trial [40].

Several limitations of this study have to be discussed: First, the cross-sectional design does not allow to draw conclusions on the longitudinal and causal association between the sciatic nerve’s FA and sAF. Another important limitation of this study is that the sample size does not allow multivariate analysis to rule out all potential confounding factors in this study. Nonetheless, to minimize the influence of potential confounders, we matched individuals and controls for age, BMI, gender, and renal function and conducted partial correlation analysis to control for potential confounders. Furthermore, sAF levels can be influenced and falsified through multiple factors: These include the intake of AGE-rich food, smoking, the application of skin creams, vasoconstriction or vasodilatation of the skin as well as the status of skin pigmentation. Although participants underwent all exams in fasting state, it cannot be ruled out that short term adjustments of diet or smoking habits influenced the measurements of sAF. However, our study population was of Caucasian ethnicity, which can be attributed to the demography of the city of Heidelberg, and study participants were instructed not to use skin care products before the measurement of sAF. Ultimately, we cannot preclude the temporary alterations of skin blood flow or previous application of skin care products may have partially influenced the measurement of sAF.

In summary this study provides novel insights into the relationship between nerve structure, intradermal AGE accumulation, and vascular health in individuals with type 2 diabetes, especially those with distal symmetric polyneuropathy. It was found that in individuals with DSPN, a lower fractional anisotropy of the sciatic nerve, indicating diminished structural nerve integrity, correlated with higher skin autofluorescence and increased vascular markers like pulse wave velocity and high-sensitivity troponin T, underscoring the link between nerve damage and vascular dysfunction. Subsequently, in the context of current research, the results of our study allow to assume that the level of skin AGEs may serve as an indicator and predictor of diabetes related complications such as DSPN. Further, the results underline that peripheral nerve damage is associated to hyperglycemia induced AGE deposition and cardiovascular risk factors such as micro- and macroangiopathy. However, additional research is needed to determine if intradermal AGE deposition accurately reflects AGE accumulation in peripheral nerves and their microcirculation, and whether this accumulation directly causes nerve damage. Further, the findings from this study endorse the need for more research into AGE-related pathways implicated in the development of DSPN, along with additional clinical trials assessing substances that inhibit or reduce AGEs for DSPN treatment or prevention.

## Data Availability

Some or all datasets generated during and/or analyzed during the current study are not publicly available but are available from the corresponding author on reasonable request.
